# Antimicrobial Resistance and Molecular Characterization of *Staphylococcus aureus* Recovered from Cows with Clinical Mastitis in Dairy Herds from Southeastern Brazil

**DOI:** 10.3390/antibiotics11040424

**Published:** 2022-03-23

**Authors:** Gustavo Freu, Tiago Tomazi, Antonio F. S. Filho, Marcos B. Heinemann, Marcos V. dos Santos

**Affiliations:** 1 Milk Quality Research Laboratory (Qualileite), Department of Nutrition and Animal Production, University of São Paulo, Pirassununga, São Paulo 13635-900, Brazil; gustavofreu@usp.br; 2 Technical Services, Merck Animal Health, Kenilworth, NJ 07033, USA; tiago.tomazi@merck.com; 3 Department of Preventive Veterinary Medicine and Animal Health, School of Veterinary Medicine and Animal Science, University of São Paulo, São Paulo 05508-900, Brazil; antoniosouzafilho@gmail.com (A.F.S.F.); marcosbryan@usp.br (M.B.H.)

**Keywords:** antimicrobial resistance, clinical mastitis, spa-typing, *Staphylococcus aureus*

## Abstract

*Staphylococcus aureus* is a contagious pathogen frequently associated with bovine mastitis in Brazil. Molecular characterization of *Staph. aureus* isolated from affected mammary quarters of cows with clinical mastitis (CM) can provide data on epidemiological behavior of this pathogen and antimicrobial susceptibility (AMS) assessment at the genotypic level. This study genotypically characterized *Staph. aureus* isolates recovered from cows with CM and determined the association of genotypes and AMS. A total of 84 *Staph. aureus* strains identified from affected mammary quarters of cows with CM in 13 dairy herds from Southeastern Brazil were submitted for susceptibility testing to 10 antimicrobials using the technique of minimal inhibitory concentration. The same isolates were also genotyped using the spa-typing methodology. Results showed a high genotypic similarity between the *Staph. aureus* isolates within and between herds, which were categorized as resistant to most antimicrobials, especially to β-lactam antibiotics. In addition, differences in AMS were observed among genotypic clusters, which may affect the efficacy of antimicrobials used to treat CM in different dairy herds.

## 1. Introduction

Bovine mastitis is a common disease in dairy herds causing significant economic losses to the dairy industry [1]. *Staphylococcus aureus* is a major pathogen of mastitis, which is mainly transmitted between cows by the contagious route, causing both subclinical and clinical mastitis (CM) [2,3]. Additionally, some strains comprising this species are considered a threat to public health [4].

Although the prevalence of *Staph. aureus* has been reduced in US dairy herds [5,6], this pathogen remains a frequent cause of mastitis in countries with developing dairy industry, such as Brazil [7]. Epidemiological studies carried out worldwide report prevalences of *Staph. aureus* ranging from 0 to 70.3% in dairy herds [8,9].

Molecular characterization of mastitis-causing pathogens allows monitoring of specific features at the strain level, such as transmission routes and antimicrobial resistance. Among available molecular typing techniques, spa-typing is a reproducible and affordable method, which differs from multi-locus sequence typing (MLST) method, in that spa-typing involves the typing of a single locus [10,11,12]. These characteristics make spa-typing a useful technique for assessing the genetic diversity of *Staph. aureus* isolated from bovine mastitis [13,14].

There is a major public and animal health concern about infections caused by antimicrobial-resistant *Staph. aureus* [13,15,16]. Antimicrobial therapy remains an important strategy for treatment of CM caused by *Staph. aureus*, especially in dairy farms treating all cases of the disease regardless of pathogen. However, the bacteriological cure rates of CM caused by *Staph. aureus* after antimicrobial therapy is usually low [2] and depends on characteristics associated with the cow, mastitis-causing strain, environment conditions and therapeutic protocol used to treat the disease [17]. These factors may result in greater use of antimicrobials in dairy herds, which in consequence, can contribute to the increase in *Staph. aureus* resistance to available commercial drugs. 

Strains of *Staph. aureus* causing mastitis resistant to multiple antimicrobials have been identified in several countries [18,19], including Brazil [20]. However, there are limited data on antimicrobial resistance of *Staph. aureus* isolated from cows with CM at the genotypic level. Assessing the strain-level antimicrobial resistance of *Staph. aureus* causing CM in Brazil can advance the pharmaco-epidemiology knowledge about this pathogen, especially considering the overuse of antimicrobials reported for treatment of CM in that country [21].

Furthermore, the assessment of genetic distribution and resistance profile of *Staph. aureus* can help in understanding the dispersion of circulating clones causing CM, contribute to the use and development of effective interventions and management practices, and help in the identification of possible transmission routes [22]. Therefore, the objectives of this study were: (a) to characterize the genotypic diversity of *Staph. aureus* isolated from affected mammary quarters of cows with CM in 13 dairy herds; and (b) determine the association between *Staph*. *aureus* resistance to antimicrobials according to the genetic similarity of strains.

## 2. Results

### 2.1. Descriptive Results and Spa-Typing

Of isolates initially cryopreserved (*n* = 112), 28 were not included in this study because of the following reasons: 11 did not grow when re-cultured for antimicrobial susceptibility testing, 8 were contaminated, 6 were isolated from repeated cases of CM within 14 days, and 3 did not have the species confirmed by MALDI-TOF MS (i.e., MALDI score < 2.0). Therefore, 84 *Staph. aureus* isolates from 13 dairy herds were evaluated in this study (Table 1). Detailed characteristics of the herds are described elsewhere [7].

Selected isolates were identified from affected mammary quarters of cows with CM housed in free stalls (*n* = 28, 33.0%), compost bedded pack barn (26.2%; *n* = 22), and grazing paddocks (40.5%; *n* = 34). In addition, 58 isolates (69.0%) were identified from mild cases of CM, whereas 22 (26.2%) were from moderate cases and only one (1.2%) from a severe case of CM. For 3 isolates (3.6%), the CM severity was not registered at the CM diagnosis (Table 1).

**Figure 1:**
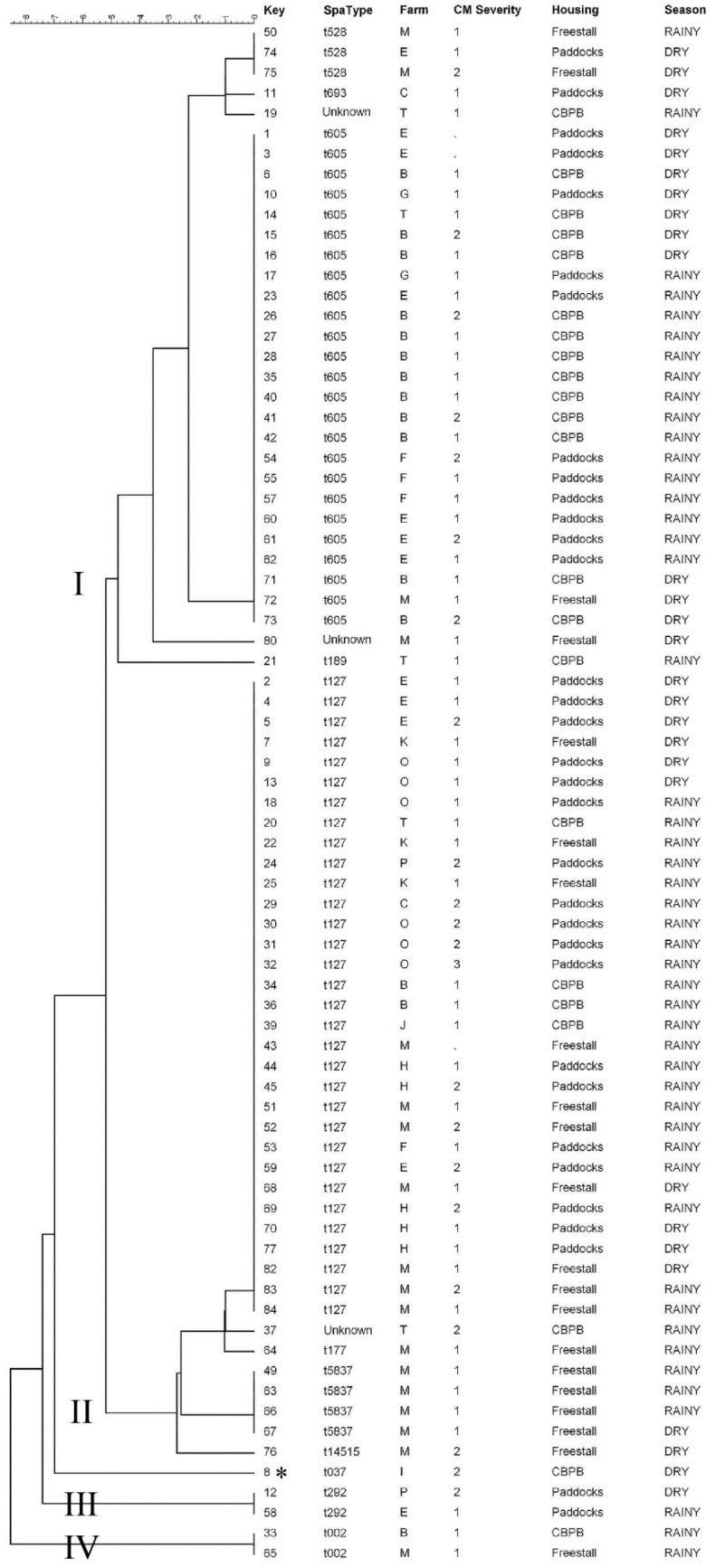
Clusters of spa-typing profiles of 76 *Staph. aureus* recovered from affected mammary quarters of cows with clinical mastitis in 13 dairy herds in Brazil. The data is presented according to the spa-typing classification, farm of origin, severity score of clinical mastitis, housing system used in the herd of origin, and season of clinical mastitis identification. For three isolates, the spa-type was unclassified (named here as “unknown”), and one isolate (* *t*037) was not assigned to any cluster.

The DNA of eight isolates did not amplify during PCR, and therefore, the dendrogram was generated with the spa-typing profiles of 76 isolates (Figure 1). Four clusters (I, II, III and IV) were identified according to the genetic similarity of *Staph. aureus* isolates. Cluster I was composed of 32 isolates and had four spa-types (*t*). Cluster II had 39 isolates and had four spa-types. Clusters III and IV had only two isolates each and shared the same spa-types within clusters. Two isolates from cluster I and one isolate from cluster II did not have the spa-type recognized by the molecular method and were named in this study as “unclassified”. In addition, one isolate (named here as t037) had a lower level of similarity in comparison to other isolates and was not assigned into clusters. (Table 1; Figure 1).

**Figure 2:**
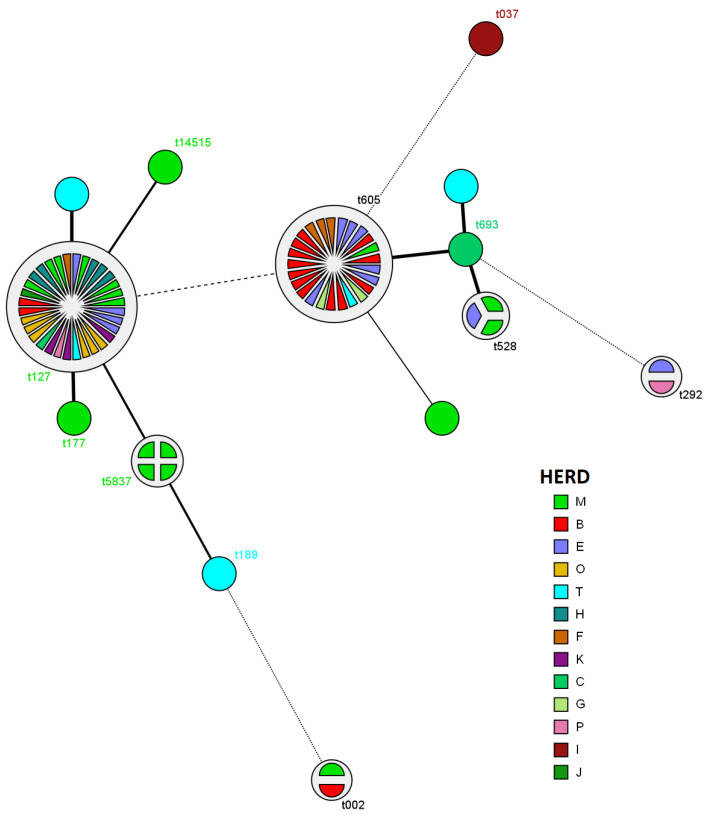
Minimum spanning tree (MST) showing 76 *Staphylococcus aureus* isolates recovered from affected mammary quarters of cows with clinical mastitis and genotyped using the spa-typing method. Each node represents one spa-type, and the corresponding spa-type is given beside the node. The distribution of spa-types within herds are represented by colors. Lengths ≤ 1 are represented by dotted lines while lengths >1 by solid lines. Three isolates did not have spa-type recognized by the molecular method.

Of the 76 isolates with spa-typing results, the most prevalent types were t127 (*n* = 32; 42.1%) and t605 (*n* = 25; 32.9%; Figure 1 and Figure 2). Based on the MST analysis, a high within- and between-herds similarity was observed among the isolates. For example, 12 of 15 isolates (80%) identified from cows with CM in the herd B pertained to the spa-type t605. Furthermore, 83.3% of *Staph. aureus* isolated in the herd E were classified as spa-types t605 (*n* = 6) and t127 (*n* = 4; Figure 2).

### 2.2. Overall Antimicrobial Susceptibility Testing

MIC results and the characterization of all *Staph. aureus* isolates as susceptible or resistant to antimicrobials are shown in Table 2. More than 50.0% of isolates were resistant to erythromycin (54.8%) and penicillin (60.7%). On the other hand, high susceptibility to sulfadimethoxine (100.0%) and oxacillin (88.0%) was observed between isolates. For the remaining antimicrobials, the susceptibilities were: cephalothin (77.4%), ceftiofur (69.0%), penicillin + novobiocin (60.7%), pirlimycin (61.9%), ampicillin (51.2%) and tetracycline (51.2%).

### 2.3. Antimicrobial Susceptibility Testing of Genotypic Clusters

Because of the low frequency of isolates in the clusters III and IV, the antimicrobial susceptibility was compared only between clusters I and II (Table 3). *Staph. aureus* isolates belonging to cluster I were more likely to be resistant to ampicillin than isolates from cluster II (OR = 0.27; CI: 0.09–0.87; *p* = 0.03). In addition, isolates belonging to cluster I tended to be more resistant to penicillin (*p* = 0.07) and less resistant to cephalothin (*p* = 0.09) when compared to isolates from cluster II.

For the remained antimicrobials evaluated, there was no significant difference (*p* > 0.05) in antimicrobial susceptibility between clusters.

## 3. Discussion

*Staph. aureus* remains an important cause of mastitis in dairy herds around the world that causes significant losses to the dairy industry [26]. This study evaluated the genotypic diversity and antimicrobial resistance of *Staph. aureus* isolated from affected mammary quarters of cows with CM in 13 dairy herds in Brazil. Our results showed high genetic similarity in protein A gene between *Staph*. *aureus* isolated from cows with CM within and among selected herds. In addition, resistance was observed to most antimicrobials evaluated, except for sulfadimethoxine.

The *Staph. aureus* strains enrolled in our study showed variable frequencies of resistance to the antimicrobials studied, with more than 50.0% of the isolates resistant to erythromycin (54.8%) and penicillin (60.7%). Similar to our results, a recent meta-analysis evaluating data from different countries reported that mastitis-causing *Staph. aureus* had the highest frequencies of resistance to penicillin and erythromycin among several antimicrobials tested in the studies selected for the systematic review [27]. The mechanisms by which *Staph. aureus* develops resistance to antimicrobials are variable and well-studied [26,28,29]. However, one of the possible factors triggering the resistance of mastitis-causing *Staph. aureus* may be overuse of antimicrobials to treat mastitis. A recent study reported a high frequency of antimicrobial use for treatment of CM in dairy herds in Brazil [21], which included the 13 herds from which our strains originated. According to the latter study, intensive therapeutic protocols such as combined and extended therapy are broadly used for treatment of CM in Brazil, which may explain the in vitro resistance of *Staph. aureus* strains observed in the present study.

All isolates in our study were susceptible to sulfadimethoxine, which was higher than reported in another study using the same method as we used to test the antimicrobial susceptibility of mastitis-causing *Staph. aureus* (75%; [30]). It is important to mention that direct comparisons between studies can be problematic, as the use of antimicrobials (quantitatively and qualitatively) in different regions can be affected by factors such as the incidence and etiology of CM in the enrolled herds, cost of therapy, and commercially available antimicrobials for treating mastitis. For example, there is no label use of sulfonamides for treatment of mastitis in the US, while in Brazil this antimicrobial is widely used (e.g., by intramammary and/or systemically route; [21]). High resistance to sulfadimethoxine has been reported in *Staph. aureus* strains isolated in Latin American herds [27], which may be associated with the high use of this antimicrobial in dairy herds for treatment of infectious diseases. However, it is important to mention that sulfadimethoxine is a long-lasting sulfonamide not commonly recommended for the treatment of *Staph. aureus* causing mastitis. Although the resistance of *Staph. aureus* to sulfadimethoxine is reported in the literature, our results showed the opposite (100% sensitivity). It is important mentioning that in vitro studies may yield different results than in vivo studies, which may be associated with specific *Staph. aureus* features such as its capacity to invade the mammary gland cells, and form biofilm and micro-abscesses [2,31], which do not occur in tests performed *in vitro.*

Twelve percent of isolates in our study were resistant to oxacillin. Our result is higher than the 2.9% reported in North American dairy herds [30], but similar to that found in Chinese herds (12%; [32]). Although oxacillin is not approved for mastitis treatment in the US and Brazil, resistance to this antibiotic has been used as an indicator of methicillin-resistant *Staph. aureus* (MRSA) [32,33]. The frequency of MRSA isolated from milk samples from cows with mastitis is historically low [33], although an increase in the frequency of oxacillin-resistant bacteria has been reported over the past 50 years [27]. Implementing preventive measures to control mastitis caused by *Staph. aureus* can help prevent the spread of MRSA in dairy herds, once MRSA constitutes a risk factor for public health [34]. This can be especially important for Brazilian dairy herds where contagious pathogens are still a problem.

Our study found high genotypic similarity between *Staph. aureus* strains within and between selected herds. The majority of isolates were assigned to one of the two most prevalent clusters (I and II), in which two spa-types (t127 and t605) had the highest frequencies of identification. As extensively reported in previous studies, *Staph. aureus* is considered a major pathogen of mastitis, which is mainly transmitted by the contagious route [17,27,35]. The high genotypic similarity between strains in our study corroborate the latter statement. Similar and genetically close-related strains observed in our study may be the most prevalent *Staph. aureus* subspecies in selected herds, as they share the same geographic region (i.e., southeastern Brazil). According to Pacha et al. [36], common strains among dairy herds may indicate a dispersal source of *Staph. aureus*. In this case, herd biosecurity to reduce or eliminate the introduction of new strains and the movement of animals and the rotation of workers between farms are recognized as risk factor for spreading of *Staph. aureus* pathogenic strains [36,37]. As most of the herds in our study shared the same geographical region, the aforementioned events may have occurred, although it was not assessed herein.

According to the antimicrobial susceptibility comparison between the genotypic clusters, *Staph. aureus* belonging to cluster I were 0.27 times more resistant to ampicillin, and tended to be more resistant to penicillin and less resistant to cephalothin when compared to isolates belonging to cluster II. β-lactam antibiotics are among the most frequently used antimicrobials in dairy cows for treatment and/or prevention of mastitis [14,27,38]. In dairy herds in Brazil, β-lactam antibiotics are also among the most used compounds for treatment of CM, especially drugs administered systemically [21]. The overuse of β-lactam antibiotics may be one of the reasons associated with the overall resistance of *Staph. aureus* isolates to cephalosporins and penicillins in our study. In addition, differences in susceptibility observed between clusters may be associated with the presence of genetic differences among the isolates [39]. The higher resistance to ampicillin and penicillin in cluster I may be associated with genes expressing resistance to antibiotics of the penicillin class, which may be less frequent in strains belonging to cluster II. Genetic characterization studies evaluating the presence of specific resistance genes of strains can elucidate those matters, although such assessment was beyond the scope of our study.

The better understanding of the epidemiology of *Staph. aureus* genotypes can advance strategies to reduce the spread of infections. Some strains comprising the *Staph. aureus* species are considered a threat to public health [4]. Therefore, continuous antimicrobial susceptibility surveillance of *Staph. aureus* recovered from dairy cows is needed and can provide valuable information about changes in the resistance pattern of this pathogenic bacterium over time. The differences in antimicrobial susceptibility observed in our study between *Staph. aureus* strains to specific antimicrobials used for mastitis control highlight the aforementioned. Therefore, strains presenting different responses to antimicrobials can vary the treatment outcomes within and between dairy herds. 

The authors acknowledge that the number of isolates and herds evaluated in the current study is limited, however, our results can serve as reference for further large-scale studies evaluating genetic traits associated with antimicrobial resistance and presence of specific genetic features associated with the resistance of *Staph. aureus* causing bovine mastitis. 

## 4. Materials and Methods

### 4.1. Staphylococcus Aureus Isolates

The isolates were identified in a previous study characterizing CM in 20 dairy herds of Southeastern Brazil [7]. During the latter study, 141 cases of CM were identified as caused by *Staph. aureus* using the techniques described in the National Mastitis Council guidelines [40], and 112 were cryopreserved (−80 °C) during the study period. Twenty-nine isolates were not cryopreserved because the species identification was based on a single colony, which prevented us from carrying out additional analyses to identify the species due to insufficient material to perform subcultures. Of the stored isolates, 84 were selected for this study based on the following inclusion criteria: (a) have pure microbiological growth at re-cultivation (i.e., no contamination), (b) isolated from a new case of CM (i.e., isolates from cows with repeated cases occurring before an interval of 14 days were excluded), and (c) identified at the species level by matrix-assisted laser desorption ionization-time of flight mass spectrometry (MALDI-TOF MS), using identification score ≥ 2.0.

### 4.2. Antimicrobial Susceptibility Testing

The antimicrobial susceptibility of *Staph. aureus* isolates was performed according CLSI guidelines [24,25] and tested using a broth microdilution panel containing antimicrobials used for treatment of mastitis (Sensititre product CMV1AMAF, TREK Diagnostics, Cleveland, OH). The tests were performed according to the manufacturer’s recommendations and a total of 10 antimicrobials were evaluated: penicillin (0.12–8.0 μg/mL), ampicillin (0.12–8.0 μg/mL), oxacillin (2.0–4.0 μg/mL), cephalothin (2.0–16.0 μg/mL), ceftiofur (0.5–4.0 μg/mL), penicillin + novobiocin (1.0/2.0–8.0/16.0 μg/mL), erythromycin (0.25–4.0 μg/mL), pirlimycin (0.5–4.0 μg/mL), tetracycline (1.0–8.0 μg/mL) and sulfadimethoxine (32.0–256.0 μg/mL). Strains from the American Type Culture Collection (ATCC; *Escherichia coli* 25,922 and *Staph. aureus*, 29,213) were assessed as quality controls.

The inoculum preparation and standardization of bacterial suspensions were performed as described by Tomazi et al. [41], with slight modifications. After preparation, 100 μL of the standardized bacterial inoculum was transferred in a tube containing 11 mL of Mueller-Hinton broth cation adjusted (pH = 7.3 ± 1; BD, Sparks, MD, USA) and vortexed by 10 s. Then 50 μL of bacterial suspension was added to each well of the Sensititre^®^ panels, which were incubated at 37 °C for 20–24 h and read using the Sensititre^®^ manual viewer (TREK Diagnostic Systems, LLC, Cleveland, OH, USA).

The lowest concentration of antimicrobials capable of inhibiting 50% (MIC_50_) and 90% (MIC_90_) of *Staph. aureus* isolates were determined for each antimicrobial. The isolates were characterized as susceptible, intermediate or resistant according to the CLSI guidelines [24,25]. Isolates characterized as intermediates were interpreted as resistant.

### 4.3. Spa-Typing

Prior genomic DNA extraction, a single colony was inoculated to 1.5 mL of Brain Heart Infusion broth and incubated at 37 °C for ~24 h. The suspension was centrifuged (15000× *g* for 2 min; Mikro 200R, Hettich, Germany), the supernatant was discarded and the DNA was extracted using the Illustra bacteria genomicPrep Mini Spin Kit^®^ (GE Healthcare, Buckinghamshire, United Kingdom) following the manufacturer’s guidelines. The quality of the extracted DNA was assessed by measuring the absorbances at 260 nm and 280 nm using a NanoDrop 2000 spectrophotometer (Thermo Scientific, Wilmington, DE, USA).

The repeated region of the protein A gene was amplified according to Harmsen et al. [42]. The DNA sequences were obtained with an ABI-3500 automatic sequencer (Applied Bisystems^®^, Foster, CA, USA). Spa-types were determined with the protocol recommended by the Ridom spa Server (http://www.spaserver.ridom.de; accessed on 6 July 2017) and spa-type sequences were analyzed using the spa plugin included in Bionumerics 7.6 (Applied Maths NV, Sint-Martens-Latem, Belgium).

### 4.4. Data Analysis

The software JMP PRO 13 (SAS Institute Inc., Cary, NC, USA) was used for descriptive statistics. The spa-types distribution was assessed according to the farm from which the strain was isolated, CM severity and season of CM diagnosis (dry: April to September; rainy: October to March; [7]). The severity of CM was defined as: (mild) changes only in the milk appearance; (moderate) presence of abnormal milk accompanied by changes in the udder; or (severe) combination of abnormal milk, with signs of inflammation in the udder and systemic signs [23]. 

Clustering analysis was performed based on the Dice coefficient and the unweighted pair group method with arithmetic mean (UPGMA) using BioNumerics software v. 7.6 (Applied Maths, Sint-Martens-Ladem, Belgium). The minimum-spanning tree (MST) was generated to assess the association of clustering patterns of the herd and spa-typing. MST presented is the one with the highest overall reliability score and was calculated using UPGMA associated with the priority rule and the bootstrap resampling.

Ten separate multivariate logistic regression models were created using the binary distribution of the GLIMMIX procedure of SAS version 9.4 (SAS Institute Inc., Cary, NC, USA). For each antimicrobial comprising the broth microdilution panel, a model was created to evaluate the association between the genotypic clusters and antimicrobial susceptibility (susceptible or resistant). Herd was included as random effect in all models to control for potential herd-level confounders (e.g., differences in the drug use practices) on the antimicrobial susceptibility of isolates. Statistical significance was assumed at *p* ≤ 0.05. Tendency to significance was considered if the *p*-value was between 0.05 and 0.10.

## 5. Conclusions

Molecular analysis using spa-typing showed high genetic similarity between *Staph*. *aureus* isolated from affected quarters of cows with CM within and among selected herds. Resistance was observed to most antimicrobials evaluated, except for sulfadimethoxine. Considering the parameters used to generate the clusters based on the genotypic similarity of strains, *Staph. aureus* belonging to cluster I were more resistant to penicillin-based antimicrobials (i.e., ampicillin and penicillin) compared to cluster II. On the other hand, isolates belonging to cluster I tended to be less resistant to cephalothin compared to cluster II.

## Figures and Tables

**Table 1:** Distribution of 84 *Staphylococcus aureus* isolates recovered from affected mammary quarters of cows with clinical mastitis according to spa-typing clusters, herd of origin, housing system, season, and severity score of clinical mastitis.

Variable	Categories	I		II		III		IV		Spa-Type t037		Unclassified ^1^	
		*n*	%	*n*	%	*n*	%	*n*	%	*n*	%	*n*	%
Herd	B (*n* = 15)	12	80.0	2	13.3	-	-	1	6.7	-	-	-	-
	C (*n* = 2)	1	50.0	1	50.0	-	-	-	-	-	-	-	-
	E (*n* = 12)	7	58.3	4	33.3	1.0	8.4	-	-	-	-	-	-
	F (*n* = 5)	3	60.0	1	20.0	-	-	-	-	-	-	1	20.0
	G (*n* = 2)	2	100.0	-	-	-	-	-	-	-	-	-	-
	H (*n* = 5)	-	-	5	100.0	-	-	-	-	-	-	-	-
	I (*n* = 1)	-	-	-	-	-	-	-	-	1	100.0	-	-
	J (*n* = 1)	-	-	1	100.0	-	-	-	-	-	-	-	-
	K (*n* = 3)	-	-	3	100.0	-	-	-	-	-	-	-	-
	M (*n* = 25)	4	16.0	13	52.0	-	-	1	4.0	-	-	7	28.0
	O (*n* = 6)	-	-	6	100.0	-	-	-	-	-	-	-	-
	P (*n* = 2)	-	-	1	50.0	1.0	50.0	-	-	-	-	-	-
	T (*n* = 5)	3	60.0	2	40.0	-	-	-	-	-	-	-	-
Housing ^2^	CBPB ^3^ (*n* = 22)	15	68.1	5	22.7	-	-	1	4.6	1	4.6	-	-
	Freestall (*n* = 28)	4	14.3	16	57.1	-	-	1	3.6	-	-	7	25.0
	Paddocks (*n* = 34)	13	38.3	18	52.9	2	5.9	-	-	-	-	1	2.9
Season	Rainy (*n* = 53)	18	34.0	27	50.9	1	1.9	2	3.8	-	-	5	9.4
	Dry (*n* = 31)	14	45.2	12	38.7	1	3.2	-	-	1	3.2	3	9.7
CM severity ^4^	Mild (*n* = 58)	23	39.7	25	43.1	1	1.7	2	3.5	-	-	7	12.0
	Moderate (*n* = 22)	7	31.8	12	54.5	1	4.6	-	-	1	4.6	1	4.5
	Severe (*n* = 1)	-	-	1	100.0	-	-	-	-	-	-	-	-
	No severity ^5^ (*n* = 3)	2	66.7	1	33.3	-	-	-	-	-	-	-	-

^1^ Isolates that were not identified by the spa-typing method; ^2^ Housing system of herds from which the *Staph. aureus* isolates were selected; ^3^ CBPB: Compost bedded pack barn. ^4^ Clinical mastitis severity was recorded as mild, moderate and severe according to Wenz et al. [23]; ^5^ Isolates that did not have record of CM severity.

**Table 2:** Overall antimicrobial susceptibility of 84 *Staphylococcus aureus* strains isolated from affected mammary quarters of cows with clinical mastitis in 13 Brazilian dairy herds.

Antimicrobial	Frequency (%) of *Staph. aureus* Isolates at Each Indicated MIC (μg/mL) ^1^												MIC_50_ ^2^	MIC_90_ ^3^
	0.12	0.25	0.5	1	2	4	8	16	32	64	128	256		
Ampicillin	44.1	7.1	17.9	11.9	8.3	3.6	7.1	-	-	-	-	-	0.25	4
Ceftiofur	-	-	11.9	32.1	25.0	31.0	-	-	-	-	-	-	2	4
Cephalothin	-	-	-	-	52.4	14.3	10.7	22.6	-	-	-	-	2	16
Erythromycin	-	44.0	1.2	9.5	2.4	42.9	-	-	-	-	-	-	1	4
Oxacillin	-	-	-	-	88.1	11.9	-	-	-	-	-	-	2	4
Penic + Novob	-	-	-	60.7	3.6	2.4	33.3	-	-	-	-	-	1	8
Penicillin	39.3	8.3	14.3	12.0	10.7	7.1	8.3	-	-	-	-	-	0.5	4
Pirlimycin	-	-	48.8	9.5	3.6	38.1	-	-	-	-	-	-	1	4
Sulphadimet.	-	-	-	-	-	-	-	-	1.2	0.0	1.2	97.6	256	256
Tetracycline	-	-	-	16.7	21.4	13.1	48.8	-	-	-	-	-	4	8

^1^ The light gray shading represents the susceptible zone, and the darker gray shading represents the resistant zone. Results were interpreted according to CLSI [24,25]. Interpretative criteria were based on human data (ampicillin, cephalothin, erythromycin, oxacillin, penicillin, sulfadimethoxine and tetracycline), and bovine mastitis (ceftiofur, penicillin + novobiocin and pirlimycin). The resistant category included isolates categorized as either intermediate or resistant; ^2^ MIC (μg/mL) that inhibited 50% (MIC_50_) of the isolates; ^3^ MIC (μg/mL) that inhibited 90% (MIC_90_) of the isolates.

**Table 3:** Results of the regression models comparing the antimicrobial susceptibility of 71 *Staph. aureus* strains isolated from affected mammary quarters of cows with clinical mastitis according to their genetic similarity into spa-typing clusters I (*n* = 32) and II (*n* = 39).

Antimicrobial	Resistance ^1^ (LSM ^2^, %)		Odds Ratio (95% CI ^3^)	*p*-Value	MIC_50_ ^4^		MIC_90_ ^5^	
	Cluster I	Cluster II			Cluster I	Cluster II	Cluster I	Cluster II
Ampicillin	57.4	26.8	0.27 (0.09, 0.87)	0.03	0.5	0.12	4	4
Ceftiofur	44.9	28.4	0.49 (0.17, 1.41)	0.18	1	2	4	4
Cephalothin	13.2	29.9	2.80 (0.82, 9.56)	0.09	2	4	16	16
Erythromycin	58.5	52.6	0.79 (0.26, 2.36)	0.67	1	1	4	4
Oxacillin	9.4	7.7	0.81 (0.15, 4.45)	0.80	2	2	2	2
Penicillin	75.2	49.3	0.32 (0.09, 1.11)	0.07	2	1	8	8
Penic + Novob	50.2	32.8	0.48 (0.18, 1.31)	0.15	0.5	0.25	4	8
Pirlimycin	45.8	38.4	0.74 (0.25, 2.17)	0.57	2	1	4	4
Sulfadimethoxine	92.3	95.5	1.78 (0.21, 15.27)	0.59	256	256	256	256
Tetracycline	56.3	52.2	0.85 (0.29, 2.43)	0.75	8	4	8	8

^1^ The interpretation criteria to categorize the isolates as resistant were based on guidelines of CLSI [24,25]. Interpretative criteria were based on human data (ampicillin, cephalothin, erythromycin, oxacillin, penicillin, sulfadimethoxine and tetracycline), and bovine mastitis (ceftiofur, penicillin + novobiocin and pirlimycin). The resistant category included isolates categorized as either intermediate or resistant.; ^2^ Least square means; ^3^ 95% confidence interval; ^4^ MIC (μg/mL) that inhibited 50% (MIC_50_) of the isolates; ^5^ MIC (μg/mL) that inhibited 90% (MIC_90_) of the isolates.

## Data Availability

All datasets are available from the corresponding author on reasonable request.
